# Cancer patient distress and health service use is linked with carer distress: evidence from a systematic review and meta-analysis

**DOI:** 10.1007/s00520-026-10759-y

**Published:** 2026-05-30

**Authors:** Thi N. T. Huynh, Tracey DiSipio, Louisa G. Collins, Lee Jones, Rachel E. Neale, Vanessa L. Beesley

**Affiliations:** 1 Psychedelic Medicine and Supportive Care Laboratory, QIMR Berghofer, Brisbane, Australia; 2 School of Public Health, The University of Queensland, Brisbane, Australia; 3 Viertel Cancer Research Centre, Cancer Council Queensland, Brisbane, Australia; 4 Statistics Group, QIMR Berghofer, Brisbane, Australia; 5 Cancer Aetiology and Prevention Laboratory, QIMR Berghofer, Brisbane, Australia; 6 School of Psychology, The University of Queensland, Brisbane, Australia; 7 School of Nursing, Queensland University of Technology, Brisbane, Australia

**Keywords:** Anxiety, Cancer, Carers/caregivers, Depression, Distress, Health service use, Psychological wellbeing, Meta-analysis, Systematic review

## Abstract

**Background:**

Family carers (caregivers) of cancer patients often have poor mental health, which may adversely affect patient wellbeing and health service use. We examined the evidence for this using a systematic review and meta-analyses.

**Methods:**

We systematically searched online databases for studies reporting associations between carer psychological health and either: (a) psychological health of patients with cancer they cared for; or (b) health service use in carers or patients. Studies’ risk of bias was assessed using the Joanna Briggs Institute Critical Appraisal Tool. We undertook meta-analyses to estimate pooled correlations for the most commonly reported associations between carer and patient psychological health. For associations between carer psychological health and carer and/or patient health service use, we conducted a narrative synthesis.

**Results:**

Our search identified 11,911 records, 169 of which were eligible. The majority of studies were of high-to-moderate quality. Carers who were distressed, depressed, anxious, or had poor scores in the mental component of quality of life were significantly more likely to be caring for patients with these same outcomes (pooled effect sizes ranged from 0.28 to 0.42; all *p* < 0.001). Subgroup analyses by gender, disease stage, and study quality revealed no substantial differences. Carers with poor psychological health used general practice, mental healthcare, and hospital services more frequently than those who were psychologically healthy, and the patients they cared for used more medications and had more frequent emergency presentations.

**Conclusion:**

The inclusion of carers alongside patients in early psychosocial care may improve family outcomes and reduce health service use.

**Supplementary Information:**

The online version contains supplementary material available at 10.1007/s00520-026-10759-y.

## Background

In 2020, an estimated 19.3 million people were newly diagnosed with cancer worldwide, and this number is projected to increase by 47% by 2040 [1]. There is growing recognition that family members play a fundamental role in supporting patients with cancer [2]. Partners, parents, adult children, or friends providing care to patients with cancer, alongside professionally trained and paid carers, are referred to as family carers or caregivers [2]. They perform numerous demanding tasks related to activities of daily living, management of disease- and treatment-related symptoms, administering medication, and navigating complex healthcare systems, often while facing the impending loss of their loved one [2]. In the USA, carers were found to spend an average of 33 h each week caring for cancer patients, with 32% devoting 41 or more hours per week, more than the time spent on a full-time job [3]. A study which collected data on carer time across multiple countries from 2008 to 2018 (converted to 2017 Canadian dollars) found the value of carer time varied from CAD975 to CAD19,112 per month, averaging CAD4809 per month excluding out-of-pocket costs such as travel expenses and medications [4]. These estimates highlight the substantial economic contribution of family carers in cancer care.

Family carers may assume the caring role for emotional and economic reasons, but they are unlikely to have the necessary knowledge and skills to deal with the complexities inherent in caregiving responsibilities, and generally feel unprepared for this role [2]. Additionally, they need to juggle career demands with social lives and other family responsibilities.

**Fig. 1:**
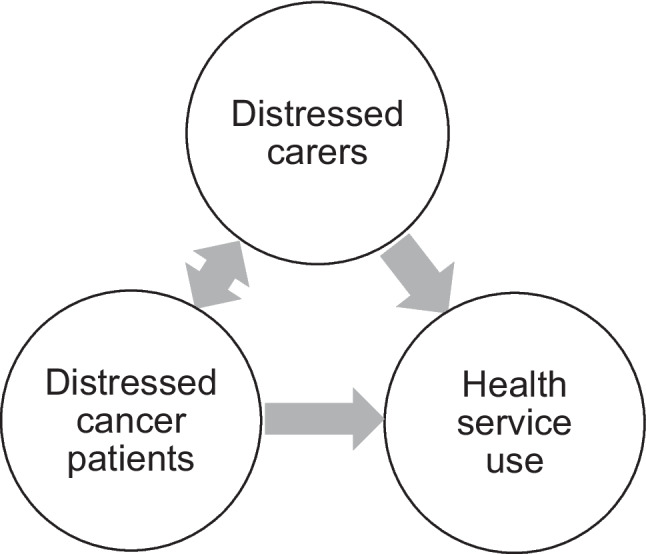
Representation of associations among carer and patient psychological health and health service use

Some family carers of cancer patients experience significant impacts on their psychological health [5, 6], including distress, anxiety, depression, fear of recurrence, and anticipatory grief. Cancer carers have higher levels of psychological distress than the general population [7] and family carers of frail elderly people or patients with other chronic diseases [8]. Consequently, family carers of cancer patients often become ‘secondary patients’ [9], seeking healthcare services to address the deleterious psychological effects of caregiving [10], leading to elevated healthcare costs [11]. Figure 1 illustrates these relationships.

Although the associations between cancer carer psychological health and patient psychological health have been widely investigated, and some systematic reviews exist [12, 13], these focused only on distress rather than specific outcomes such as anxiety and depression, and findings remain inconsistent. Furthermore, the association between cancer carer psychological health and health service use is not well understood. Thus, we conducted a systematic review and meta-analysis to summarise the current evidence. Increased knowledge about the strength and patterns of associations across specific psychological outcomes will help to determine the potential impacts of better addressing the psychological burden among carers.

## Methods

We used the Preferred Reporting Items for Systematic Reviews and Meta-Analyses (PRISMA) checklist as a framework [14] and registered the study with the International Prospective Register of Systematic Reviews (PROSPERO) prior to commencing (registration number CRD42023453347).

### Eligibility criteria

Eligible studies had to have been written in English, published in a peer-reviewed journal, and included data on the psychological health of family cancer carers and either: (a) psychological health of adults or children diagnosed with cancer; or (b) patient or carer health service/medication use. We excluded reviews, protocols, conference abstracts, qualitative studies, case studies, editorials, letters, opinion pieces, books, and theses.

### Information sources and search details

We identified potentially eligible studies via searches of the following databases: PubMed, PsychINFO, EMBASE, CINAHL, and Cochrane Library. Reference lists of all studies accepted for inclusion were scanned for any additional eligible articles.

The searches were conducted between the 20th and 27th of February 2024. The search strategy was adapted for each database (see Supplementary File S1). There was no limit on the date of publication. 

### Manuscript selection

We used Covidence (Veritas Health Innovation, Melbourne, Australia) to manage search results. After duplicates were removed, the first 5317 records were reviewed by authors TH and TD, based on the eligibility criteria. As agreement was close to 90%, TH assessed the remainder. Title/abstract screening excluded articles that were clearly not relevant. Full texts of remaining articles were then reviewed to determine their eligibility. A hierarchy of criteria for exclusion was recorded (Supplementary File S2). Any disagreements between the two reviewers regarding study inclusion were discussed and VB was consulted when necessary.

### Data collection and items

Data were extracted into Microsoft Excel 2016. TH and Tara Caterina Kwan (TCK) abstracted information from 47 papers, including: study characteristics (i.e. first author, publication year, country of study, study design, study duration, time since diagnosis, numbers of carers and patients, cancer site, stage, study setting (centre-based or population-based) and inclusion criteria); participant characteristics (i.e. age, gender, relationship between carer and patient); and study outcomes (i.e. measurement instrument, psychological health outcome, health service use measure, relevant associations, and covariates). As agreement was close to 90% between TH and TCK, TH assessed the remainder. TD reviewed the data extraction for completeness and accuracy.

### Risk of bias

TH and TD independently assessed a random sample of ten articles for risk of bias using the Joanna Briggs Institute (JBI) Critical Appraisal Tool [15]. The criteria were modified according to the features of the present review (Supplementary Table 4); e.g. cohort studies required ≥ 3 months of follow-up, and ≤ 20% drop-out or reasons explained for higher drop-out to be classified as high quality. Responses to each question were as follows: yes, no, unclear, or not applicable. Discrepancies were settled by discussion between the reviewers and, when necessary, consultation with VB. We calculated the quality of each study by dividing the total number of yes responses by the total number of applicable responses (i.e. yes/no/unclear). Higher percentages indicate lower risks of bias [15]. The guidelines for applying JBI tools do not suggest cut-off thresholds for classifying studies based on their quality scores [15]. We classified studies as follows: high quality 90–100%; moderate quality 70% and 89%; low quality < 70%. As TH and TD had 90% agreement on quality, TH assessed the remaining studies.

### Summary measures

Summary measures of carer and patient distress were described as means (standard deviations), medians (interquartile ranges), or percentages. Summary measures of health service use were described as proportions. Associations between carer psychological outcomes and patient psychological outcomes and/or health service use were extracted as correlation coefficients, odds ratios, relative risks, mean differences, or beta-coefficients.

### Statistical analysis

For the associations between carer and patient psychological outcomes, we firstly used a narrative approach to synthesise findings. We then used random effects meta-analyses to calculate pooled estimates of commonly reported associations between carer and patient psychological outcomes. For longitudinal studies with repeated measures, we only included cross-sectional data, usually reported at baseline. We included studies if they reported Pearson’s or Spearman’s correlation coefficients, using adjusted estimates where available and unadjusted estimates otherwise. These were transformed into Fisher’s z estimates to facilitate the calculation of summary effects. We reported pooled estimates using Pearson’s correlation coefficient (r). Beta-coefficients were not included because they were predominantly generated using structural equation models or path models so did not represent the same type of association as simple correlation coefficients. We considered a two-tailed *p*-value ≤ 0.05 to be statistically significant. Prediction intervals were calculated. Analyses were carried out using R software, version 4.1.2 (R Foundation for Statistical Computing, Vienna, Austria).

We used the I^2^ and Cochran’s Q test to examine heterogeneity in effect sizes. We considered I^2^ values of 0–40%, 41–60%, and 61–100% as low, moderate, and high heterogeneity, respectively. Q tests with a *p*-value < 0.10 indicated significant heterogeneity. We examined the influence of carer gender (predominantly female (i.e. at least two thirds); predominantly male; mix of male and female carers), disease stage (predominantly advanced (i.e. at least two thirds); predominantly localised; mix of advanced and localised disease), and study quality (high; moderate; low) on meta-analysis results.

We used funnel plots and Egger’s tests to assess publication bias. An asymmetric funnel plot may suggest the presence of publication bias. The Egger’s test provided statistical evidence for funnel plot asymmetry, with a *p*-value < 0.10 indicating statistically significant publication bias.

For associations between carer psychological health and carer and/or cancer patient health service use, we carried out a narrative synthesis.

## Results

### Study selection

**Fig. 2:**
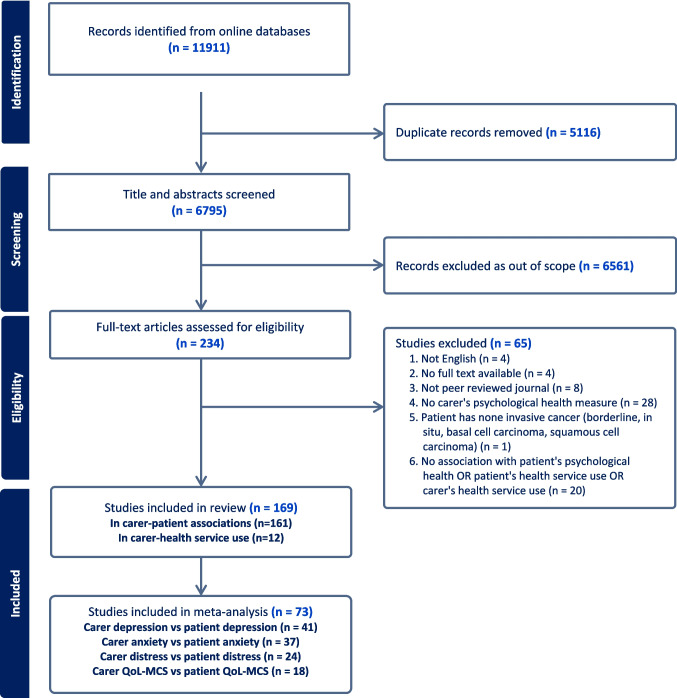
The PRISMA flow diagram for records included

We identified 11,911 records from database searches (Fig. 2). After removing duplicates, 6795 records proceeded to title and abstract screening. Of these, 234 underwent full-text review; 65 did not meet inclusion criteria, leaving 169 eligible studies.

### Characteristics of the included studies

Characteristics of the 169 studies are summarised in Supplementary Table 1. They were conducted in 31 countries, all published after 1985. Most were cross-sectional in design (*n* = 122), with some using longitudinal (*n* = 45) or case-control designs (*n* = 2). No clinical trials met inclusion criteria. The sample size ranged from 15 to 910 cancer patient-carer dyads or cancer carers. All studies included adult carers; spouses/partners of cancer patients were the exclusive carers in 44 studies, while the remaining 125 included any person providing informal care. In nearly half of the studies (47%, *n* = 81) carers were predominantly female, 42% (*n* = 70) included a mix of men and women, 7% (*n* = 11) included predominantly male carers, and 4% (*n* = 7) were unclassifiable. Most studies (90%, *n* = 153) included adult cancer patients, 5% (*n* = 8) included cancer patients aged less than 18 years, and 5% (*n* = 8) did not provide characteristics of the patient population. More than a quarter of the studies (28%, *n* = 48) included cancer patients with predominantly advanced disease, 15% (*n* = 25) with predominantly localised disease, 11% (*n* = 19) a mix of advanced and localised disease, and almost half (46%, *n* = 77) were unclassifiable.

Study quality assessments are shown in Supplementary Table 4. Briefly, 45% of the studies (*n* = 76) were classified as high quality, 48% (*n* = 81) as moderate, and 7% (*n* = 12) as low. A common reason for bias was not reporting demographic characteristics or history of having psychological conditions before cancer diagnosis among carers or patients. Many studies also failed to adjust for important confounders, including carers’ age, gender, education, and cancer stage; only 40% (*n* = 68) controlled for at least one demographic variable. However, the strength of associations between carer and patient psychological health varied across studies irrespective of whether such variables were adjusted for.

### Cross-sectional associations between carer and patient psychological health

**Fig. 3:**
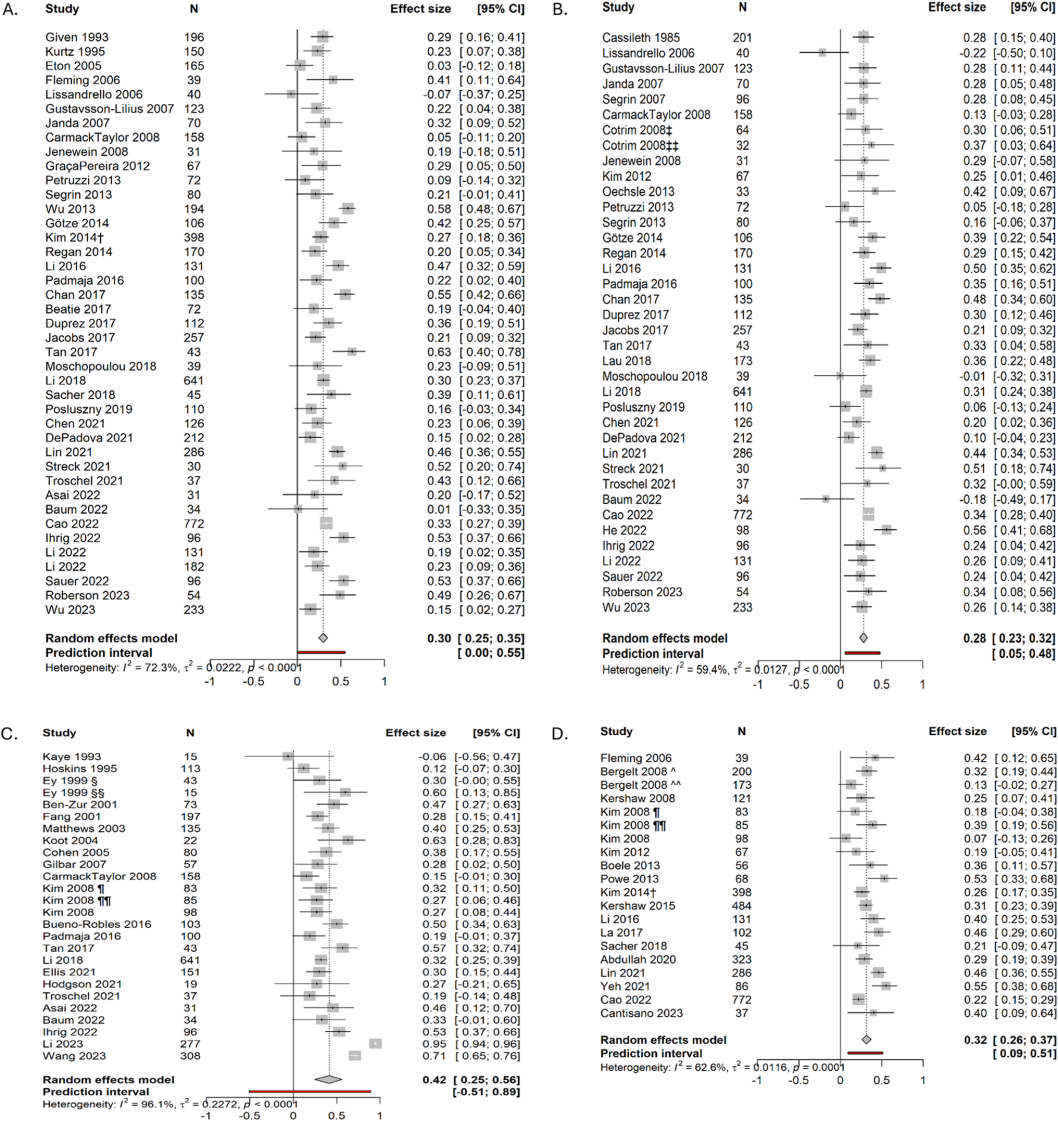
Forest plots of associations between carer depression and patient depression (**A**), carer anxiety and patient anxiety (**B**), carer distress and patient distress (**C**) and carer QoL-MCS and patient QoL-MCS (**D**). †All cancer patient-carer dyads; ‡Dyads with non-stoma patients; ‡‡Dyads with stoma patients; § Husband carers; §§ Wife carers; ¶Breast cancer; ¶¶Prostate cancer; ^Female carer; ^^Male carer

We identified 161 studies that investigated cross-sectional associations between two same or different psychological outcomes in carers and patients (Supplementary Table 2). The most commonly tested within dyad (i.e. carer-patient) associations examined the same outcomes in both members: (1) depression (*n* = 54); (2) anxiety (*n* = 51); (3) distress (*n* = 37); and (4) mental component score of quality of life (QoL-MCS) (*n* = 23). The Hospital Anxiety and Depression Scale [16], the Brief Symptom Inventory [17], the Centre for Epidemiology Scale for Depression [18], and the State Trait Anxiety Inventory [19] were most commonly used to measure distress, depression, and anxiety. Several tools were used to assess QoL-MCS, including the Medical Outcomes Study–Short Form 36 [20]/Short Form 12 [21] and the European Organisation for Research and Treatment of Cancer–Quality of life questionnaire C30 [22]/C15 [23]. Among the bivariable or multivariable estimates, 125 correlation coefficients were reported (Fig. 3), ranging from −0.22 to 0.95, of which 94 (75%) were statistically significant. We extracted 70 β-coefficients across the four associations from multivariable regression models, structural equation models or path models, ranging from −0.99 to 0.75. More than half (38/70) of the β-coefficients were statistically significant. The majority of bivariable (121/125) or multivariable (68/70) estimates were positive. There were four negative correlation coefficients and two negative β-coefficients reported from moderate quality studies, which included adolescent or young participants (median age = 32–37 years) [24, 25] or patients diagnosed with curable cancers [24, 26–28].

Other psychological outcomes reported in the literature for carers and patients were psychological burden, fear of recurrence/progression (FOR/FOP), individual quality of life domains (i.e. psychological functioning or mental health), stress, post-traumatic stress syndrome/disorder, negative affect, general mental health, general psychological health, mood disturbance, psychiatric symptoms, or anticipatory grief. Among these, the most common analyses performed included 14 studies examining associations between stress and any of QoL-MCS, depression, anxiety, distress, or FOR [29–42]. Both significant and non-significant results were reported. The strongest association was observed between carer perceived stress and patient FOR (*r* = 0.581, *p* < 0.01) [39].

### Longitudinal associations between carer and patient psychological health

Although 42 longitudinal studies employed structural equation models or path models to examine the directional associations between psychological outcomes in carers and cancer patients, half (*n* = 21) reported significant interdependence between carer and patient psychological outcomes without establishing clear directionality. Several studies (*n* = 15) indicated that poorer patient psychological outcomes preceded and predicted poorer carer outcomes; for example, anxiety in breast cancer patients at 6 weeks was found to predict carer anxiety at 6 months [43]. In contrast, a smaller number of studies (*n* = 6) suggested the opposite, whereby carer outcomes influenced patient outcomes; for example, lower QoL-MCS in carers at baseline led to lower QoL-MCS in advanced cancer patients at 3 months [44].

### Meta-analyses of associations between carer and patient psychological health

Four separate meta-analyses were performed to synthesise correlation coefficients of the four most commonly analysed relationships; 73 studies were included in at least one of the meta-analyses. We retrieved 41 associations between carer and patient depression, 38 between carer and patient anxiety, 26 between carer and patient distress, and 20 between carer and patient QoL-MCS (Fig. 3). All four meta-analyses showed moderately strong pooled effect sizes: carer/patient anxiety (*r* = 0.28); carer/patient depression (*r* = 0.30); carer/patient QoL-MCS (*r* = 0.32); carer/patient distress (*r* = 0.42) (all *p*-values < 0.001) (Table 1). While these findings indicate that poorer psychological outcomes in carers were associated with poorer psychological outcomes in patients, I^2^ ranged from 59 to 96% and Q statistics from 50 to 633 (all *p*-values < 0.001). Due to substantial between-study heterogeneity, the prediction interval for the association between carer and patient distress included zero (Fig. 3).

**Table 1:** Pooled associations of cancer carer and patient psychological outcomes

Psychological outcomes	Reports	Pooled effect size** (95% CI)	*I*^**2**^	*Q*	Egger’s B*
Carer depression–patient depression	41	0.30 (0.25, 0.35)	72.3%	144.26	0.17
Carer anxiety–patient anxiety	38	0.28 (0.23, 0.32)	59.4%	91.19	−0.76
Carer distress–patient distress	26	0.42 (0.25, 0.56)	96.1%	633.23	−2.13
Carer QoL-MCS–patient QoL-MCS	20	0.32 (0.26, 0.37)	62.6%	50.79	1.07

^*^All *p*-values > 0.05

^**^All *p*-values < 0.001

**Fig. 4:**
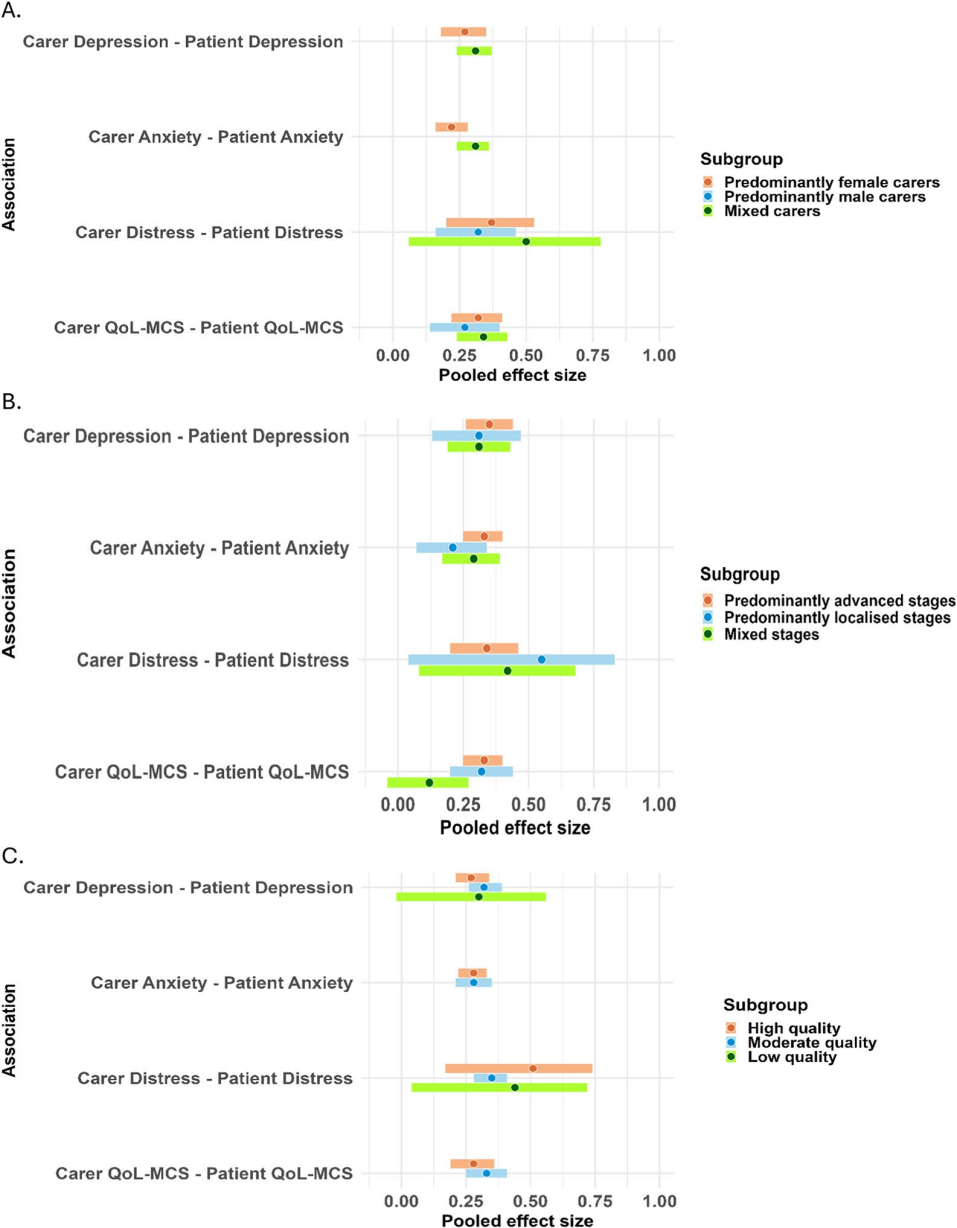
Subgroup analyses* according to (**A**) carers’ gender, (**B**) cancer stage and (**C**) study’s risk of bias. *Subgroups in which the number of effect sizes < 2 were not shown

The effect sizes across studies did not significantly vary by carer gender, cancer stage, or risk of bias (Fig. 4 and Supplementary Table 5a–c). Visual inspection of funnel plots revealed reasonable symmetry (Supplementary File S3) and Egger’s tests were not statistically significant.

### Associations between carer psychological health and carer and/or patient health service use

Supplementary Table 3 shows an overview of 12 studies that examined associations between carer psychological health and carer/patient health service or medication use. Carer psychological outcomes were depression (*n* = 6), anxiety (*n* = 4), mental health diagnosis (*n* = 3), distress (*n* = 2), psychological burden (*n* = 1), fear of recurrence (*n* = 1), anticipatory grief at baseline and prolonged grief at follow-up (*n* = 1), suicidal thoughts (*n* = 1), and self-reported overall mental health (*n* = 1). Service use outcomes included visits to health professionals to discuss mental health concerns (*n* = 3) [11, 45, 46], receipt of mental health-related treatment from psychiatrists, psychologists, counsellors, primary care providers or other providers (*n* = 8) [10, 11, 45, 47–51], visits to an emergency clinic (*n* = 1) [52], hospitalisation with an affective disorder (*n* = 1) [53], and number of medications taken (*n* = 1) [54].

The associations between carers’ psychological condition and their health service or medication use were examined in 10 studies [10, 11, 45–51, 53]. Of these, three reported that carers were 1.1 to 4.9 times more likely to visit their primary care provider or general practitioner to discuss distress following the patients’ cancer diagnosis [11, 45, 46]. Of five studies that presented odds ratios to estimate the association between carer mental health and psychosocial support services or mental health interventions [10, 11, 45, 48, 51], three produced significant results [10, 48, 51]; the strongest association was between carers’ depressed mood and antidepressant prescription or psychotherapy (OR = 5.55, *p* < 0.001) [48]. Carers with prolonged grief [45] or FOR [47] were not significantly more likely to receive mental health support than those without these diagnoses. Two studies found a significant association between carers having been diagnosed with subclinical or clinical anxiety and increased access to professional psychological help [11, 49]. One study reported a 50% increased risk of hospitalisation in cancer carers with an affective disorder compared with non-cancer carers with affective disorder [53].

With regard to the association between carer psychological condition and patient health service use, one cohort study found that patients whose carers experienced consistently high-to-severe psychological burden over a 3-year period were significantly more likely to receive polypharmacy (≥ 5 medications) [54]. Additionally, a cross-sectional study reported that patients of carers who experienced depressive symptoms at least every week had a 1.5-fold increased risk of presenting to an emergency department [52].

## Discussion

Pooling evidence from 161 studies, we found that poor psychological health in cancer carers was consistently associated with an increased risk of the cancer patients they care for having poor psychological health. Further, our meta-analyses of 73 studies revealed moderate effect sizes for the four most commonly assessed associations: carer distress, depression, anxiety, and mental component score of quality of life, each correlated with the same measure in patients. There was no evidence of publication bias, and subgroup analyses revealed consistent findings across carer gender, cancer stage, and study risk of bias. Our narrative review was the first to collate the findings of other carer-patient associations, such as carer stress with patient fear of cancer recurrence, depression, or anxiety. This is also the first review of the associations between carer psychological conditions and health service use in carers themselves and in patients they care for. Not surprisingly, the strongest associations were observed between carers reporting depressive mood and receiving antidepressant prescriptions or psychotherapy. Importantly, we also found patients were significantly more likely to receive polypharmacy or have more frequent emergency clinic presentations when their carers had poor psychological health.

Our findings on distress are consistent with two meta-analyses, one including 1098 dyads and one including 2468 couples, that found a moderate correlation between patient and carer distress scores (*r* = 0.35 and *r* = 0.29, respectively) [12, 13]. However, distress is a non-specific emotional state, often reflecting a normal reaction to a difficult situation, while depression and anxiety are diagnosable mental health conditions (e.g. via DSM-5 or ICD-11 criteria). Our meta-analysis builds on the existing meta-analyses by examining specific associations between cancer carer and patient self-reported symptoms of depression and anxiety, which are disorder-specific constructs. Although these measures do not provide formal diagnoses, they screen for probable cases using cut-off thresholds that quantify symptom severity. While there is some conceptual and empirical overlap between distress and symptoms of depression and anxiety, distress is a broader, non-specific construct often used as a first-pass indicator and may resolve with general support, psychoeducation, or coping strategies. In contrast, measures of depression and anxiety offer more precise characterisation of mental health symptoms and are more likely to indicate the need for higher-intensity interventions, such as psychological therapy, medication, or both [55, 56].

We acknowledge that associations between carer and patient poor psychological health may operate as a bi-directional process, whereby changes in one member’s psychological state influence the other, potentially leading to reciprocal deterioration over time. Our narrative synthesis of longitudinal findings supports this, indicating that the direction of effect can vary, with evidence for both carer-to-patient and patient-to-carer influences. The findings align with interdependence theory, which recognises that individuals within a dyad can influence each other’s emotions, behaviours, cognition, and outcomes [57]. Accordingly, changes in one member’s psychological state may be mirrored in the other [57]. Providing early psychological support to carers, particularly around the time of patient diagnosis, may therefore help disrupt the emergence of these reciprocal effects and mitigate downstream impacts on both members of the dyad [58]. Current practice guidelines emphasise the need for inclusion of family carers in psychosocial care [59]. Access to such support would support a better overall caregiving experience and mitigate the feeling of unpreparedness and impending loss [59]. However, limited interventions appear to be available currently for family carers in clinical practice. Further, many clinical trials of cancer carer interventions have demonstrated effectiveness in reducing carer distress [60], yet few have reported their effects on patient outcomes [61]. Future research should move beyond observational studies of associations and interventions focused solely on carer outcomes to examine the impact of carer interventions on downstream patient outcomes and potential healthcare cost savings, thereby better informing policy decisions about resource allocation.

To our knowledge, this is the first systematic review to examine the association between cancer carers’ psychological health and health service use in both carers and the patients they support. The finding that cancer carers with psychological issues have greater use of general practice and mental health providers is not unexpected. However, compared with non-cancer carers, they are more likely to require hospital-based care if they have an affective disorder, such as major depression, indicating that cancer-specific demands may be more complex, or insufficiently managed in community settings. Moreover, patients whose carers experience frequent depressive episodes are more frequently seen in emergency departments and are more likely to experience polypharmacy. These patterns point to broader challenges for health systems. Providing psychosocial support requires additional resources and may drive increased use of healthcare. However, if mental health conditions emerge or worsen, the associated escalation in care needs and service utilisation could be substantially greater; consequently, the downstream costs of not addressing psychological needs early may exceed those associated with timely intervention. Conversely, maintaining the mental health of family carers may yield important downstream benefits. For example, it may enable the patients they care for to remain in the community longer, reducing the need for frequent hospital admissions [62]. It may also enable carers to remain in paid employment, reducing financial strain, while continuing caregiving responsibilities [63]. Consistent with this, previous research has emphasised the need for policies that support family carers in the workplace, including flexible work arrangements, changes to employment status, and access to work-based support services to manage emotional stress [63]. Investing in resources that support carers’ mental well-being may therefore improve health outcomes for carers and the patients they care for and ease financial pressure on health systems, although the evidence for these downstream effects remains limited.

Collectively, these findings underscore the need for routine distress screening and integrated supportive care models early and throughout the disease course that assess and support the psychological wellbeing of both cancer patients and their carers, rather than treating them in isolation. This may be most effectively delivered by cancer nurses, who have ongoing contact with patients and carers and can identify emerging psychological needs, or, in cases of advanced disease, by palliative care teams. Doing so may improve outcomes and reduce the long-term burden on healthcare systems.

While this comprehensive meta-analysis provides robust evidence of the interdependent nature of the patient–carer relationship, the findings should be interpreted with caution due to several limitations. Most studies included were observational and cross-sectional. Among the longitudinal studies, there was substantial heterogeneity in outcomes and timing of assessments, and only a small number established clear directionality, with inconsistent results. As such, causal influences cannot be drawn, and the direction of effect remains uncertain, although existing evidence suggests they may operate in either direction. Additionally, it was not possible to disentangle whether observed dyadic interdependence reflects biological effects of cancer, psychological impacts of caregiving, or other factors such as assortative mating or shared genetic vulnerabilities, particularly given that many carers are spouses or family members. Moreover, the pooled associations were based on both unadjusted and adjusted estimates. Heterogeneity was high and was not explained by carers’ gender, cancer stage or study quality. It is possible that other factors, such as carers’ age, culture, relationship type, socioeconomic status, time since cancer diagnosis, or prior mental health history, could influence the size of the observed associations. Differences in the way information was reported, or insufficient information, hindered our ability to conduct subgroup analyses, underscoring the need for improved reporting standards. A myriad of scales and cut-offs used to examine health outcomes in carers and patients limited the possibility to make comparisons between studies. The prediction interval for the pooled effect size of the association between carer and patient distress overlapped with zero, highlighting the uncertainty of this finding. Future studies should be context-specific, aiming to understand and reduce heterogeneity rather than simply confirm the mean effect. Evidence linking carer psychological health to patient health service use was also limited, with few studies, high heterogeneity, and varying adjustment for confounders. Further research is needed to clarify these associations, as demonstrating potential economic benefits may support the routine integration of carer support into cancer care. Finally, some relevant studies may not have been identified owing to our review only including English publications with available full-texts.

## Conclusion

In conclusion, informal caregiving is a cornerstone of cancer care, and carers who experience psychological morbidity are significantly more likely to be caring for a cancer patient who also has psychological issues. However, the causality of this relationship remains unclear, and there may be bi-directional effects between carer and patient psychological health. Irrespective of this, carers’ poor psychological health has direct implications for public health sustainability, given the increased health service use by both carers and patients. Our findings emphasise the importance of health systems providing early support for cancer carers, ideally at the time of cancer diagnosis, to improve family unit outcomes and reduce the burden on professional healthcare provision.

## Supplementary Information

Below is the link to the electronic supplementary material.

ESM1 Supplementary File S1 - search strategies (DOCX 15.1 KB)

ESM2 Supplementary File S2 - hierarchy of exclusion (DOCX 12.8 KB)

ESM3 Supplementary File S3 - funnel plots (DOCX 263 KB)

ESM4 Supplementary table 1,2,3 - Data extraction (XLSX 334 KB)

ESM5 Supplementary table 4 JBI_Quality_Assessment (XLSX 33.8 KB)

ESM6 Supplementary table 5 -Subgroup analysis-3 subgroups (DOCX 3.16 MB)

## Data Availability

The data that support the findings of this study are available from the corresponding author upon reasonable request.
