# Supplementary material for: Cancer patient distress and health service use is linked with carer distress: evidence from a systematic review and meta-analysis
Source: Support Care Cancer. 2026 May 30;34(6):594. doi: 10.1007/s00520-026-10759-y (PMC13222297; doi:10.1007/s00520-026-10759-y)
Supplement: Supplementary file 1 — Supplementary File S1 - search strategies (DOCX 15.1 KB) [file 520_2026_10759_MOESM1_ESM.docx]

**Search strategy**

The search strategy which was adapted for each database included a combination of medical subject headings (MeSH) terms in PubMed, Cochrane Database Library and CINAHL, or Emtree in EMBASE, or major subject headings in PsychoINFO and keywords related to the presence of cancer, carer, psychological outcomes, patient wellbeing and health system outcomes.

1. Search strategy in PubMed:
2. ("carer*"[Title] OR "caregiver*"[Mesh] OR "family member*"[Title] OR "relative*"[Title] OR "partner*"[Title] OR "spouse*"[Title])
3. (cancer[Title] OR oncology[Title] OR Neoplasm*[Mesh] OR tumour*[Title] OR tumor*[Title] OR melanoma*[Title]) OR glioma [Title] OR glioblastoma [Title])
4. ("distress"[Title] OR "anxiety"[Title] OR "depression"[Title] OR "emotional”[Title] OR "psychological”[Title] OR "psychosocial”[Title] OR "adjustment"[Title] OR "fear”[Title] OR "grief"[Title] OR "Quality of Life/psychology"[Mesh] OR "Stress, Psychological "[Mesh] OR "Caregiver Burden"[Mesh])
5. (“health service use”[Title/Abstract] OR “health care utilization”[Title/Abstract] OR “health care utilisation”[Title/Abstract] OR “Primary care visit*”[Title/Abstract] OR “Health professional visit*” [Title/Abstract] OR “Emergency room visit*”[Title/Abstract] OR “hospital admission*”[Title/Abstract] OR “patient care”[Title/Abstract] OR “Psychological care”[Title/Abstract] OR “medication*”[Title/Abstract] OR “Hospitalisation*”[Title/Abstract] OR "Hospitalization*"[Mesh] OR "length of stay"[Mesh] OR "Health Care Cost*"[Mesh] OR "Costs and Cost Analysis"[Mesh] OR "Direct Service Cost*"[Mesh] OR "Hospital Cost*"[Mesh] OR "Drug Cost*"[Mesh] OR "Delivery of Health Care/economics"[Mesh])

The four groups was combined as (#1 AND #2) AND (#3 OR #4).

1. Search strategy in EMBASE:

(carer*:ti,ab OR Caregiver/exp OR 'family member*':ti,ab OR relative*:ti,ab OR partner*:ti,ab OR spouse*:ti,ab)
AND
(cancer:ti,ab OR oncology:ti,ab OR 'malignant neoplasm'/exp OR tumour*:ti,ab OR tumor*:ti,ab OR melanoma*:ti,ab)
AND
(distress:ti,ab OR anxiety:ti,ab OR depression:ti,ab OR 'emotional well-being'/exp OR 'fear of recurrence':ti,ab OR 'anticipatory grief':ti,ab OR 'Quality of Life'/exp OR 'mental stress'/exp OR 'Caregiver Burden'/exp)
AND
('hospital admission'/exp OR Hospitalization/exp OR 'hospital utilization'/exp OR 'bed occupancy':ti,ab OR 'hospital bed utilization'/exp OR 'Health Care Cost'/exp OR 'Cost Benefit Analysis'/exp OR 'Hospital Cost'/exp OR 'Drug Cost'/exp OR 'Cost of Illness'/exp

1. Search strategy in CINAHL:

((TI carer* OR AB carer*) OR (MH Caregivers+) OR (TI "family member*" OR AB "family member*") OR (TI relative* OR AB relative*) OR (TI partner* OR AB partner*) OR (TI spouse* OR AB spouse*))
AND
((TI cancer OR AB cancer) OR (TI oncology OR AB oncology) OR (MH Neoplasms+) OR (TI tumour* OR AB tumour*) OR (TI tumor* OR AB tumor*) OR (TI melanoma* OR AB melanoma*))
AND
((TI distress OR AB distress) OR (TI anxiety OR AB anxiety) OR (TI depression OR AB depression) OR (TI "emotional well-being" OR AB "emotional well-being") OR (TI "fear of recurrence" OR AB "fear of recurrence") OR (TI "anticipatory grief" OR AB "anticipatory grief") OR (MH "Quality of Life"+) OR (MH "Stress, Psychological"+) OR (MH "Caregiver Burden"+))
AND
((TI "hospital admission" OR AB "hospital admission") OR (MH Hospitalization+) OR (TI "bed occupancy" OR AB "bed occupancy") OR (MH "Health Care Costs"+) OR (MH "Costs and Cost Analysis"+) OR (MH "Cost-Benefit Analysis"+) OR (MH "Direct Service Costs"+) OR (MH "Hospital Costs"+) OR (MH "Drug Costs"+) OR (MH "Cost of Illness"+) OR (MH "Delivery of Health Care"+))

1. Search strategy in PsychoINFO:

((TI carer* OR AB carer*) OR (MH Caregivers+) OR (TI "family member*" OR AB "family member*") OR (TI relative* OR AB relative*) OR (TI partner* OR AB partner*) OR (TI spouse* OR AB spouse*))
AND
((TI cancer OR AB cancer) OR (TI oncology OR AB oncology) OR (MH Neoplasms+) OR (TI tumour* OR AB tumour*) OR (TI tumor* OR AB tumor*) OR (TI melanoma* OR AB melanoma*))
AND
((TI distress OR AB distress) OR (TI anxiety OR AB anxiety) OR (TI depression OR AB depression) OR (TI "emotional well-being" OR AB "emotional well-being") OR (TI "fear of recurrence" OR AB "fear of recurrence") OR (TI "anticipatory grief" OR AB "anticipatory grief") OR (MH "Quality of Life"+) OR (MH "Stress, Psychological"+) OR (MH "Caregiver Burden"+))
AND
((TI "hospital admission" OR AB "hospital admission") OR (MH Hospitalization+) OR (TI "bed occupancy" OR AB "bed occupancy") OR (MH "Health Care Costs"+) OR (MH "Costs and Cost Analysis"+) OR (MH "Cost-Benefit Analysis"+) OR (MH "Direct Service Costs"+) OR (MH "Hospital Costs"+) OR (MH "Drug Costs"+) OR (MH "Cost of Illness"+) OR (MH "Delivery of Health Care"+))

1. Search strategy in Cochrane Database Library:

(carer*:ti,ab OR [mh Caregivers] OR (family NEXT member*):ti,ab OR relative*:ti,ab OR partner*:ti,ab OR spouse*:ti,ab)
AND
(cancer:ti,ab OR oncology:ti,ab OR [mh Neoplasms] OR tumour*:ti,ab OR tumor*:ti,ab OR melanoma*:ti,ab)
AND
(distress:ti,ab OR anxiety:ti,ab OR depression:ti,ab OR "emotional well-being":ti,ab OR "fear of recurrence":ti,ab OR "anticipatory grief":ti,ab OR [mh "Quality of Life"] OR [mh "Stress, Psychological"] OR [mh "Caregiver Burden"])
AND
((hospital NEXT admission):ti,ab OR [mh Hospitalization] OR (bed NEXT occupancy):ti,ab OR [mh "Health Care Costs"] OR [mh "Costs and Cost Analysis"] OR [mh "Cost-Benefit Analysis"] OR [mh "Direct Service Costs"] OR [mh "Hospital Costs"] OR [mh "Drug Costs"] OR [mh "Cost of Illness"] OR [mh "Delivery of Health Care"])
