# Supplementary material for: Cancer patient distress and health service use is linked with carer distress: evidence from a systematic review and meta-analysis
Source: Support Care Cancer. 2026 May 30;34(6):594. doi: 10.1007/s00520-026-10759-y (PMC13222297; doi:10.1007/s00520-026-10759-y)
Supplement: Supplementary file 2 — Supplementary File S2 - hierarchy of exclusion (DOCX 12.8 KB) [file 520_2026_10759_MOESM2_ESM.docx]

The first criteria in the list which a paper did not meet was listed as the reason for exclusion. The order was as follows:

1. Publication: not published in English;
2. Publication: not peer reviewed journal;
3. Publication: full text unavailable;
4. Carer: paid carer;
5. Patient: not actual cancer (borderline, insitu, bcc, scc...), not invasive cancer;
6. Carer’s psychological health: not reported;
7. Cancer patient’s psychological health or patient’s health care use or carer’s health service use: not reported.
