# Supplementary material for: Cancer patient distress and health service use is linked with carer distress: evidence from a systematic review and meta-analysis
Source: Support Care Cancer. 2026 May 30;34(6):594. doi: 10.1007/s00520-026-10759-y (PMC13222297; doi:10.1007/s00520-026-10759-y)
Supplement: Supplementary file 3 — Supplementary File S3 - funnel plots (DOCX 263 KB) [file 520_2026_10759_MOESM3_ESM.docx]

Funnel plots of reports regarding the associations of (A) carer depression versus patient depression, (B) carer anxiety versus patient anxiety, (C) carer distress versus patient distress and (D) carer QoL-MCS versus patient QoL-MCS.

| A.  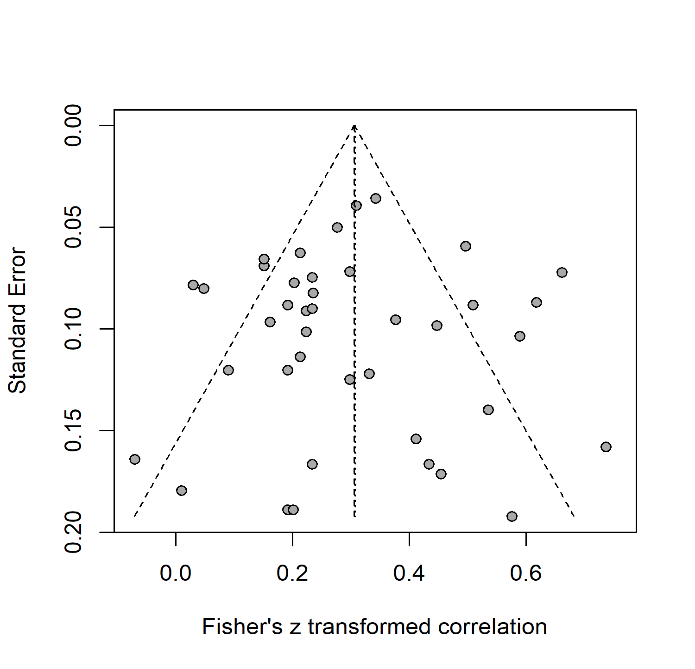 | B.  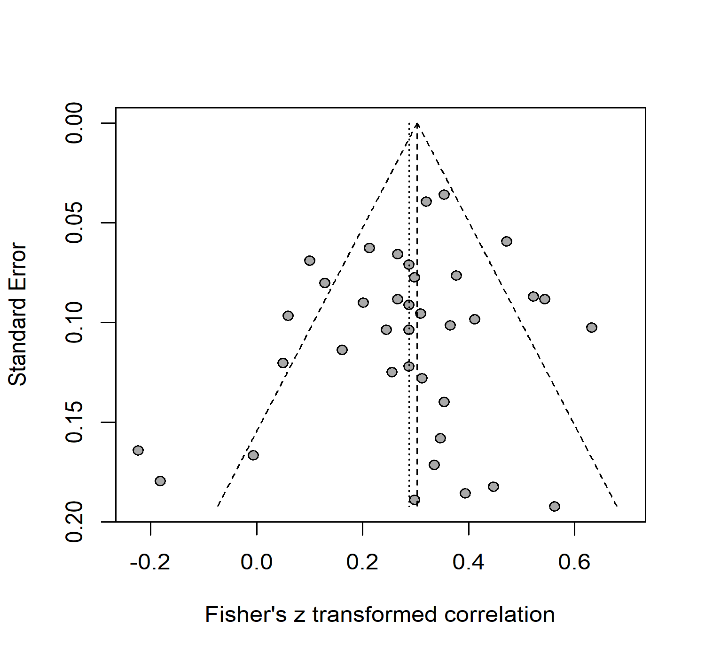 |
| --- | --- |
| C.  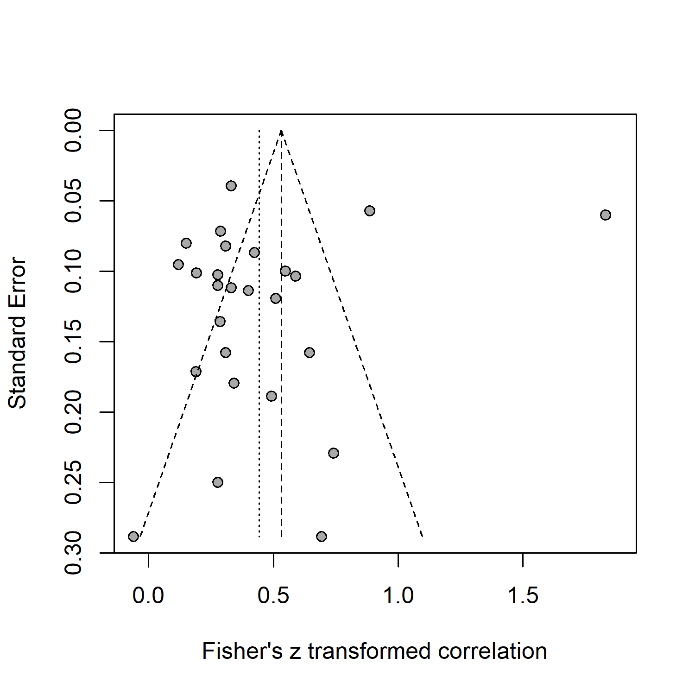 | D.  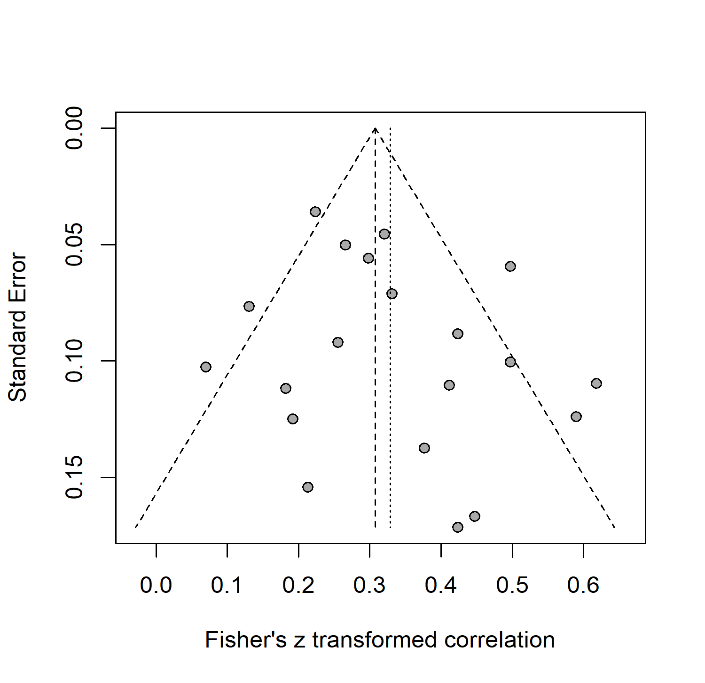 |
