# Supplementary material for: Cancer patient distress and health service use is linked with carer distress: evidence from a systematic review and meta-analysis
Source: Support Care Cancer. 2026 May 30;34(6):594. doi: 10.1007/s00520-026-10759-y (PMC13222297; doi:10.1007/s00520-026-10759-y)
Supplement: Supplementary file 6 — Supplementary table 5 -Subgroup analysis-3 subgroups (DOCX 3.16 MB) [file 520_2026_10759_MOESM6_ESM.docx]

**Supplementary table 5a.** Subgroup analyses regarding gender* for (A) Carer and Patient Depression, (B) Carer and Patient Anxiety, (C) Carer and Patient Distress, and (D) Carer and Patient QoL-MCS.

| A.  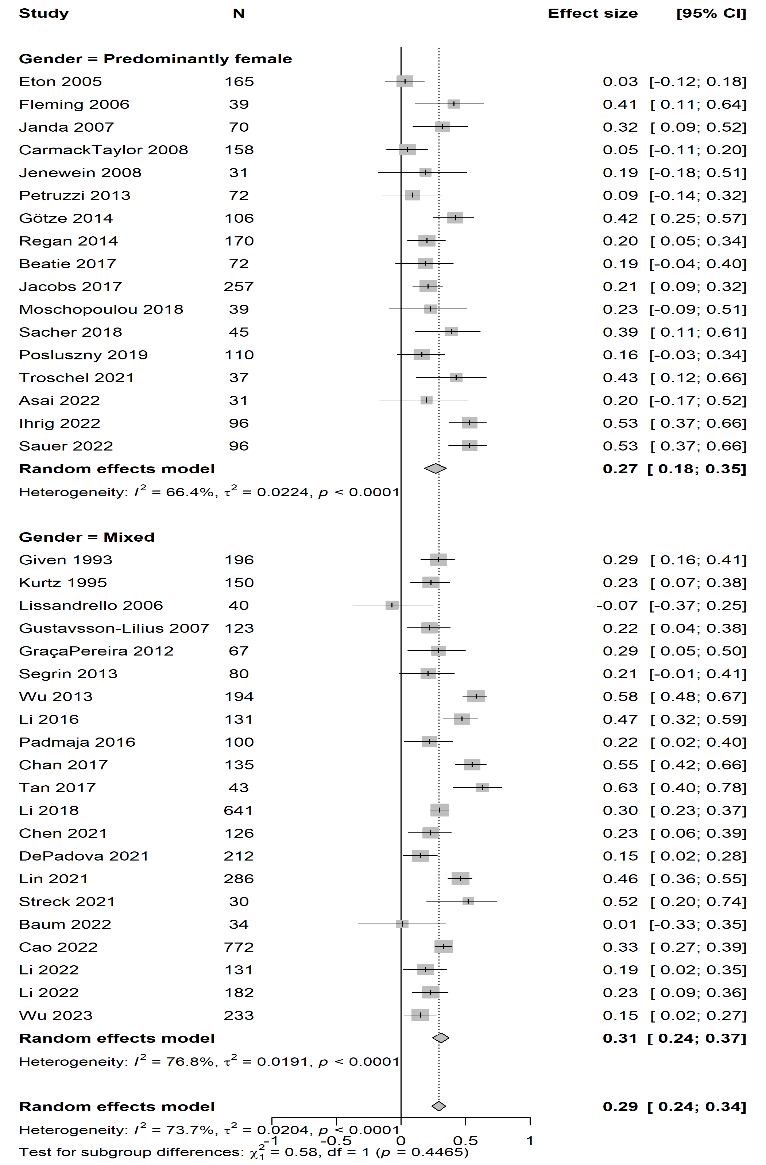 | B.  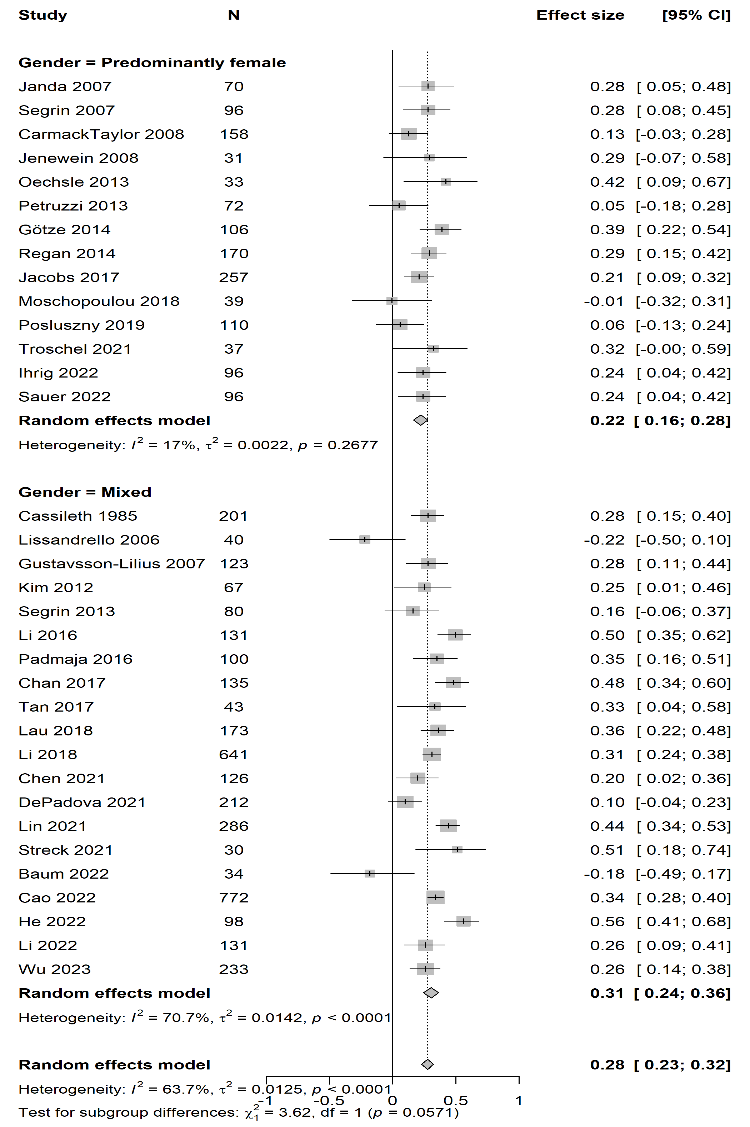 |
| --- | --- |
| C.  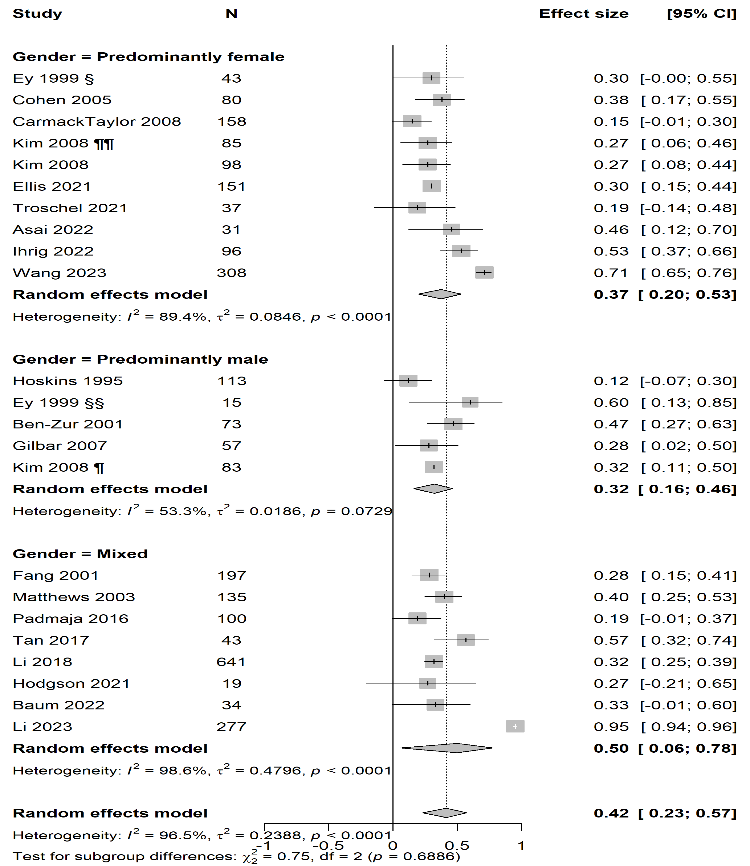 | D.  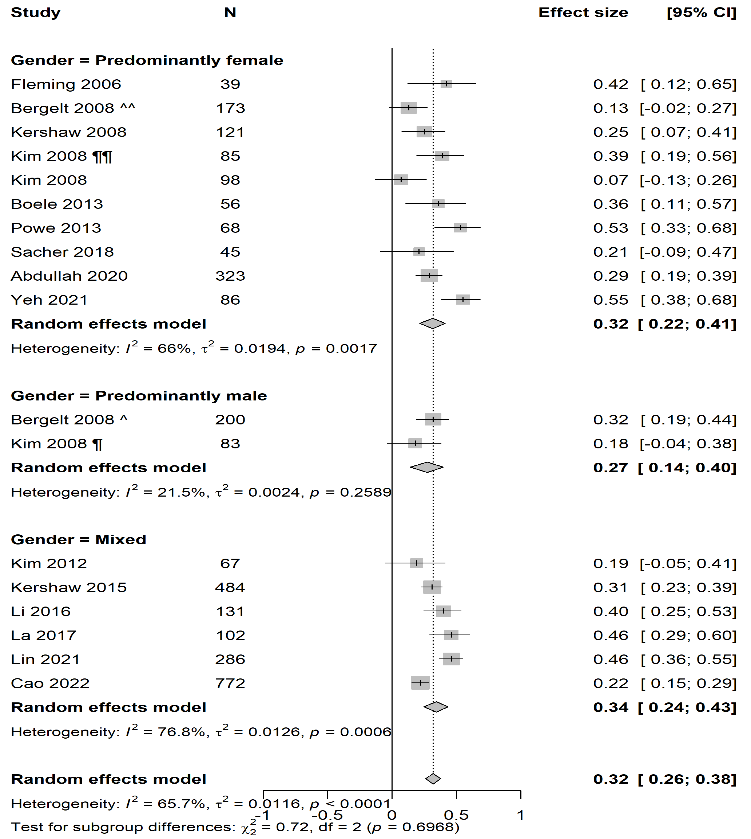 |

§ Husband carers; §§ Wife carers; ¶Breast cancer; ¶¶Prostate cancer; ^ Female carer; ^^ Male carer.

*Subgroups in which the number of effect sizes <2 were not shown.

**
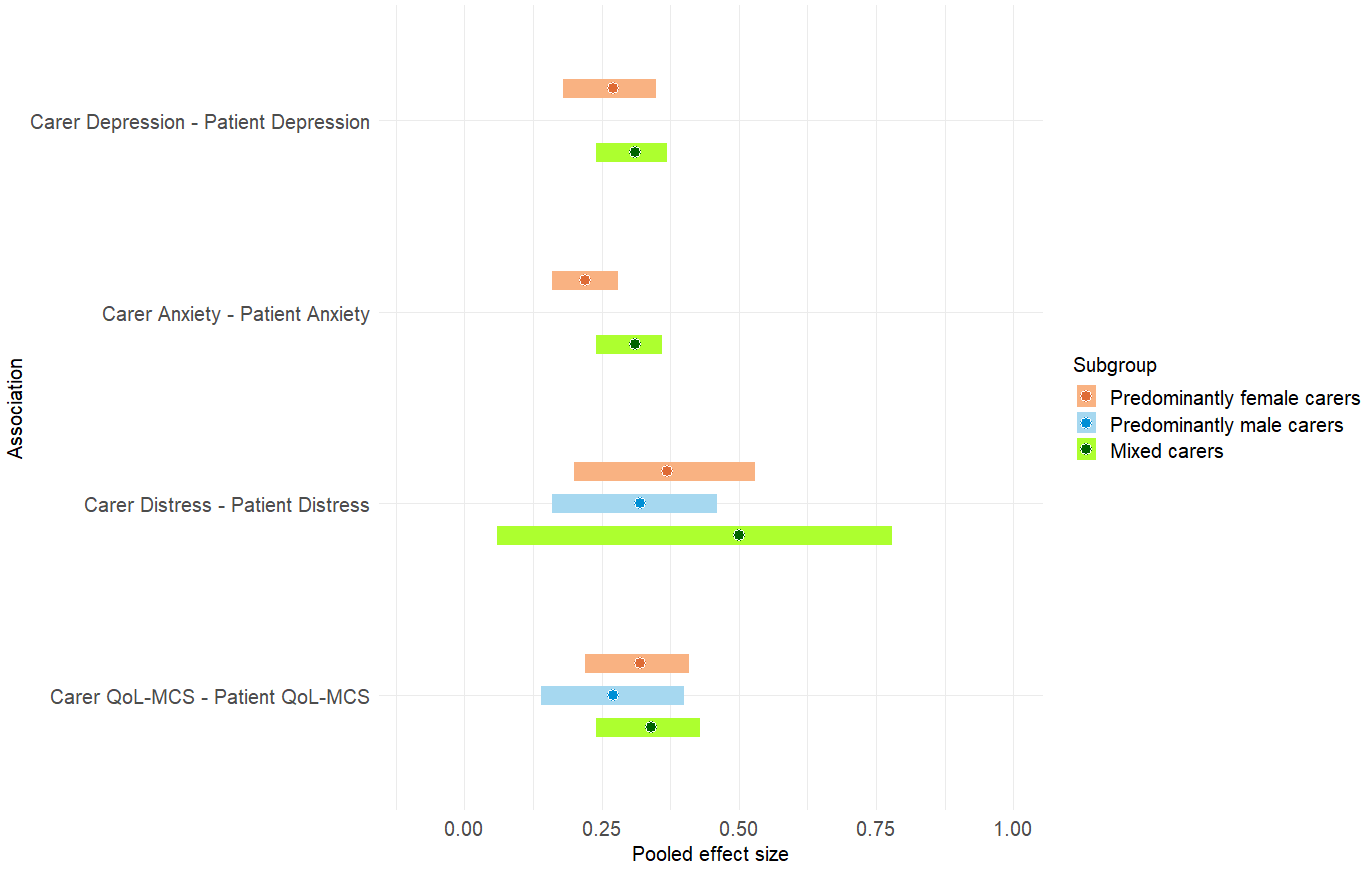
**

*Subgroups in which the number of effect sizes <2 were not shown.

**Supplementary table 5b.** Subgroup analyses regarding cancer stage for (A) Carer and Patient Depression, (B) Carer and Patient Anxiety, (C) Carer and Patient Distress, and (D) Carer and Patient QoL-MCS.

| A.  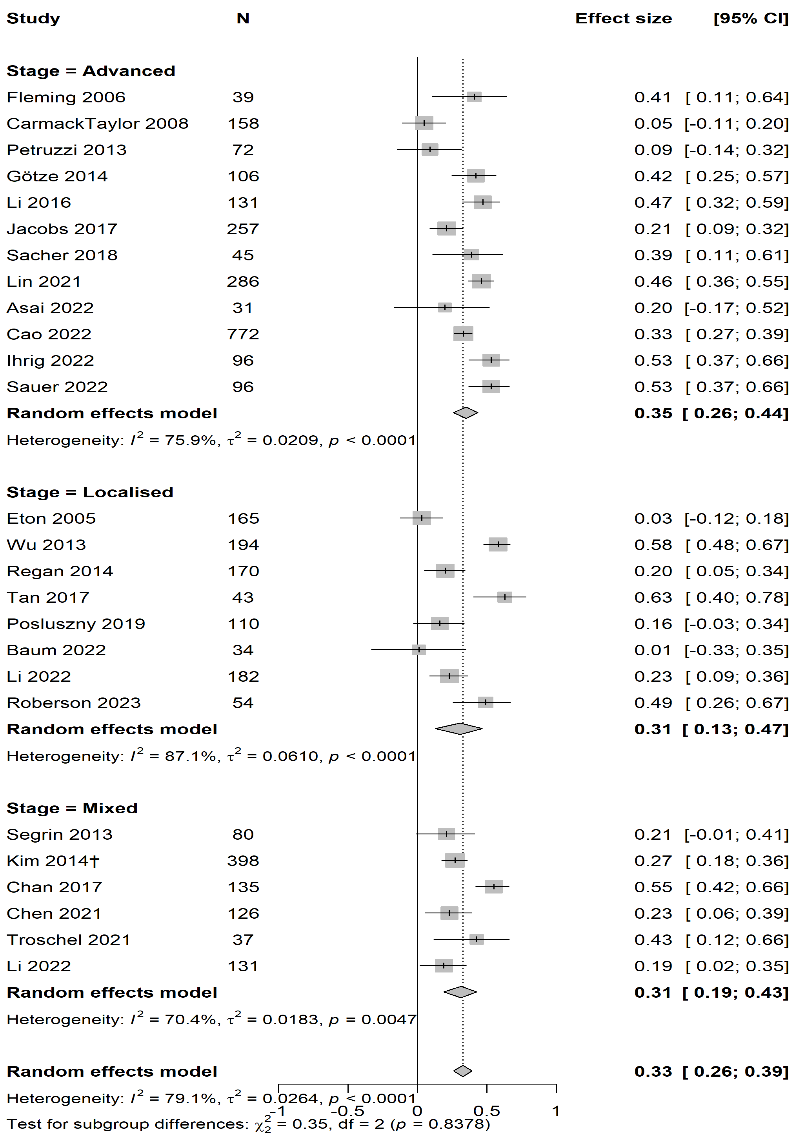 | B.  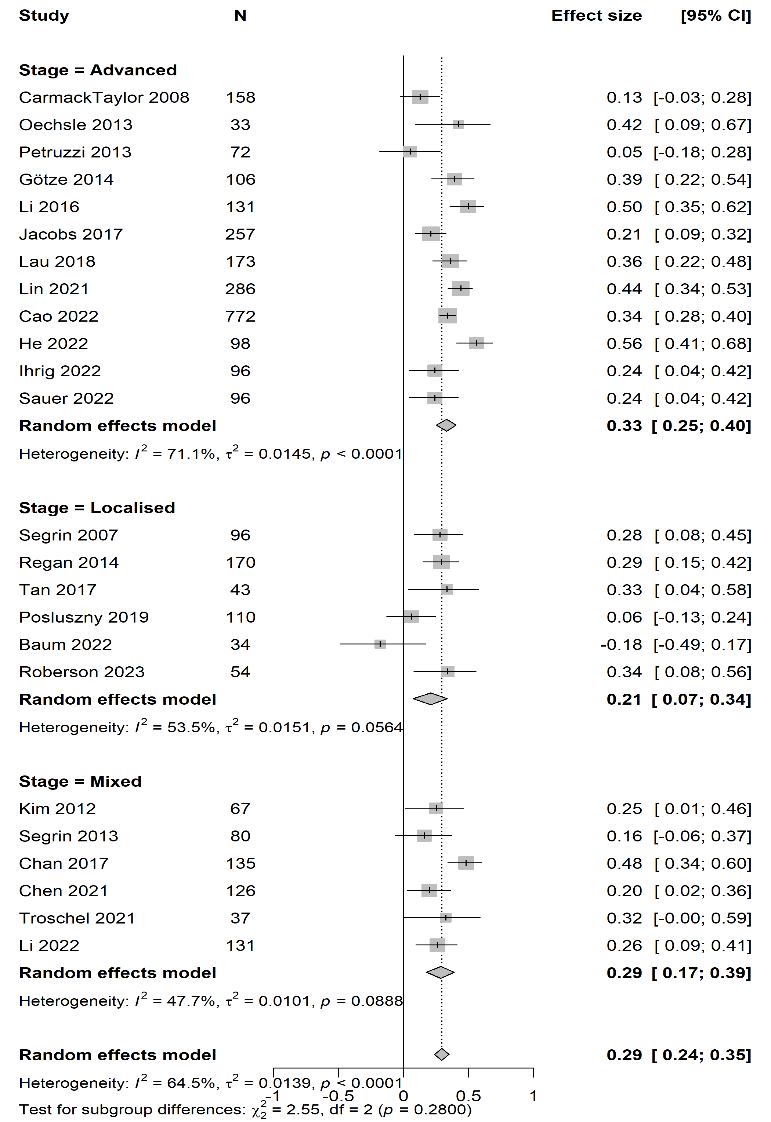 |
| --- | --- |
| C.  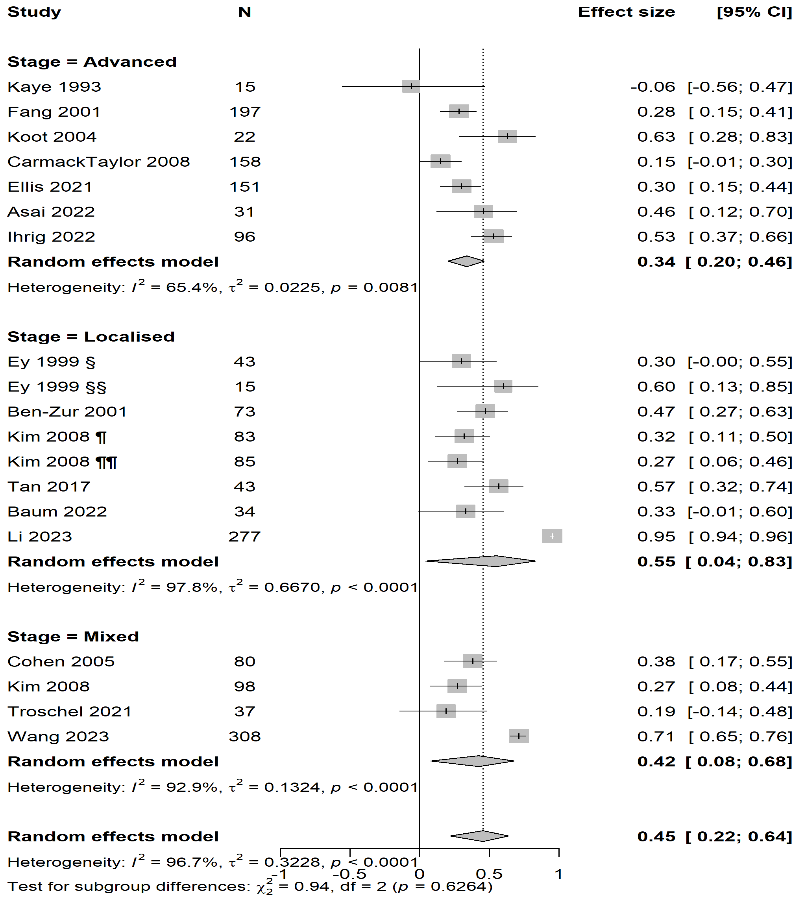 | D.  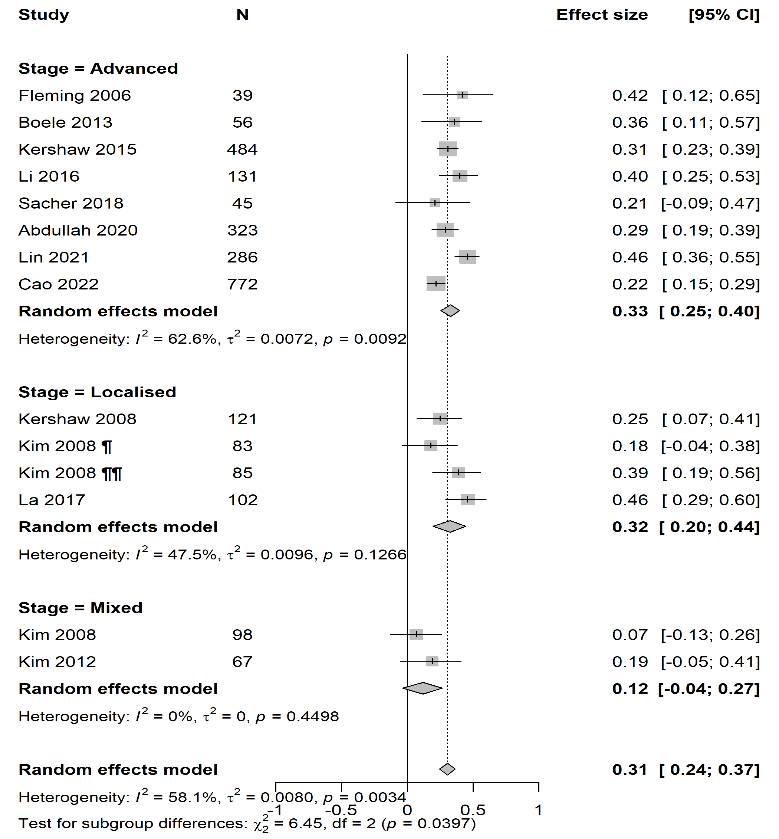 |

†All cancer patient-carer dyads; § Husband carers; §§ Wife carers; ¶Breast cancer; ¶¶Prostate cancer.

*Subgroups in which the number of effect sizes <2 were not shown.


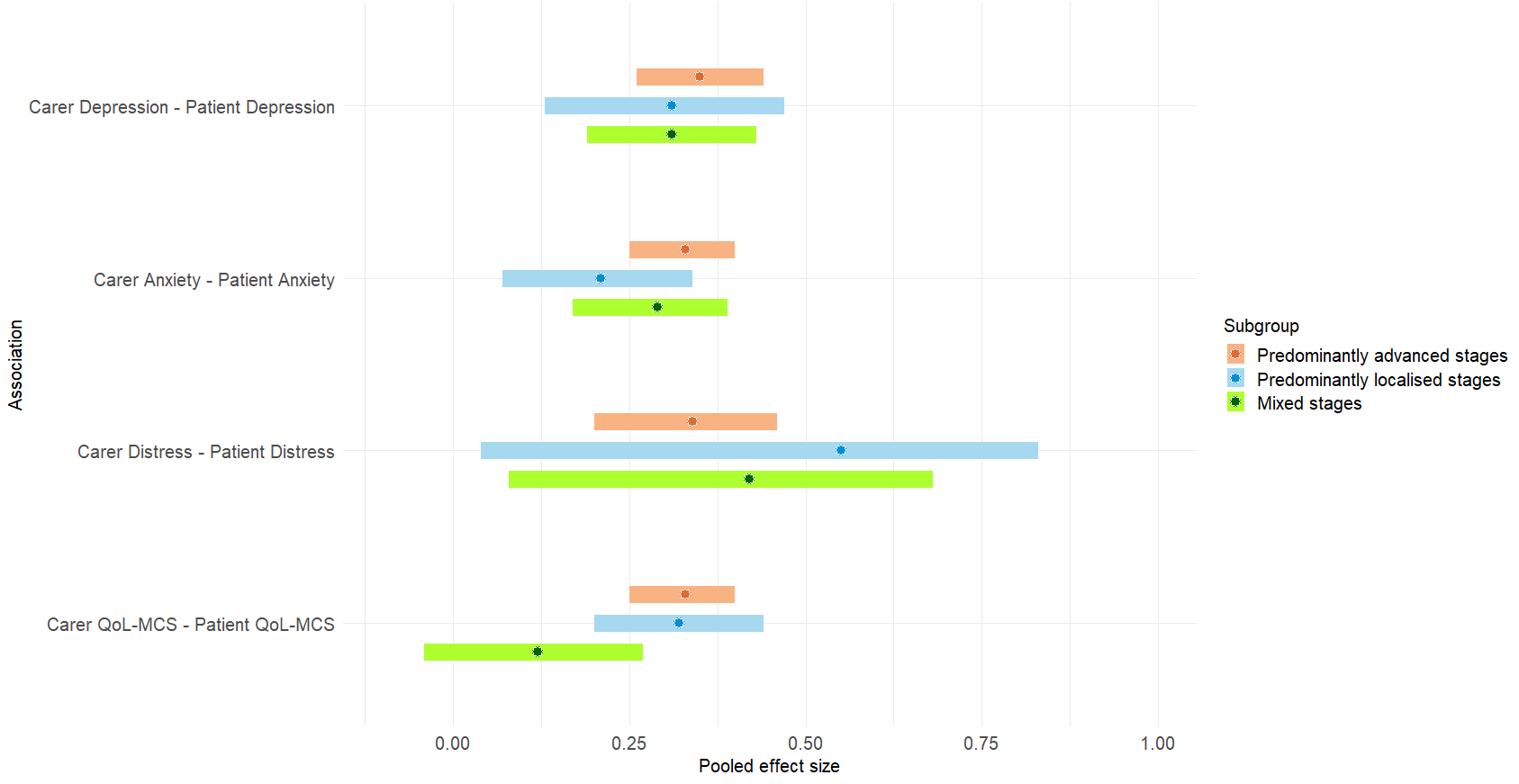


*Subgroups in which the number of effect sizes <2 were not shown.

**Supplementary table 5c.** Subgroup analyses regarding study quality* for (A) Carer and Patient Depression, (B) Carer and Patient Anxiety, (C) Carer and Patient Distress, and (D) Carer and Patient QoL-MCS.

| A.  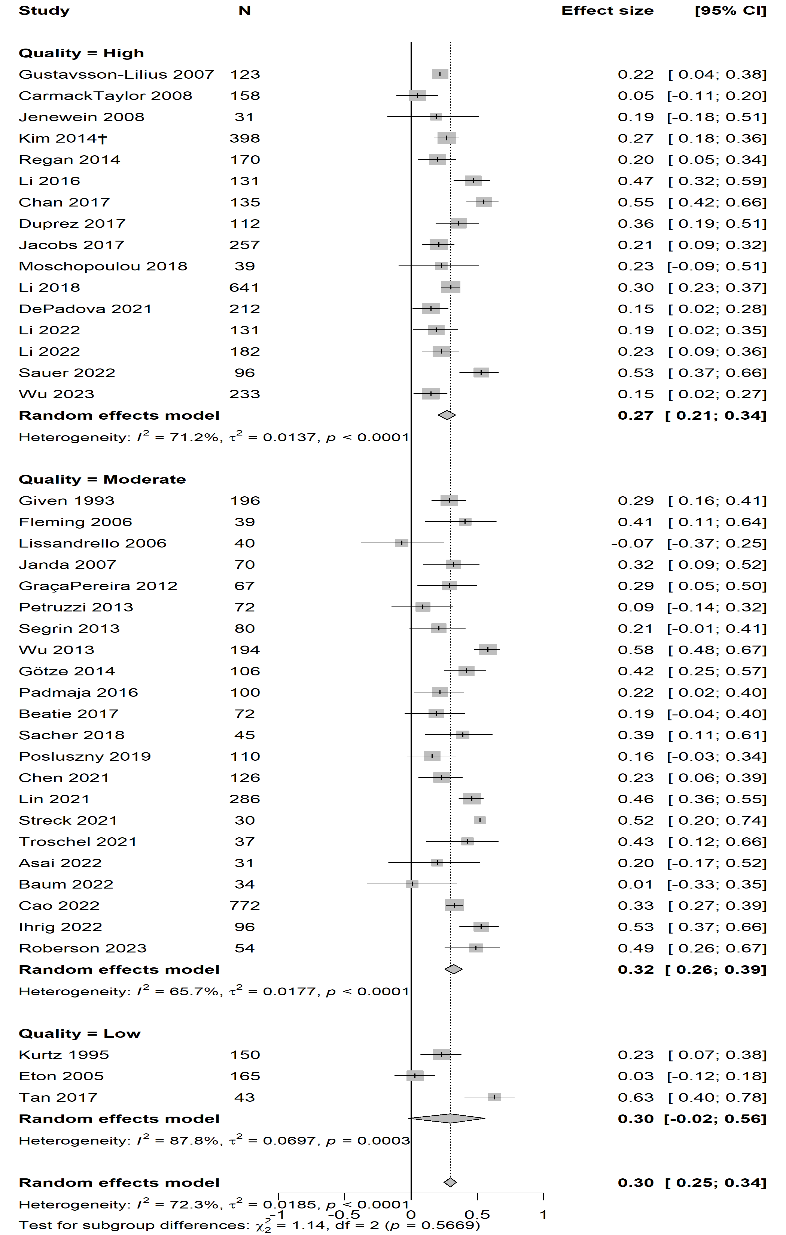 | B.  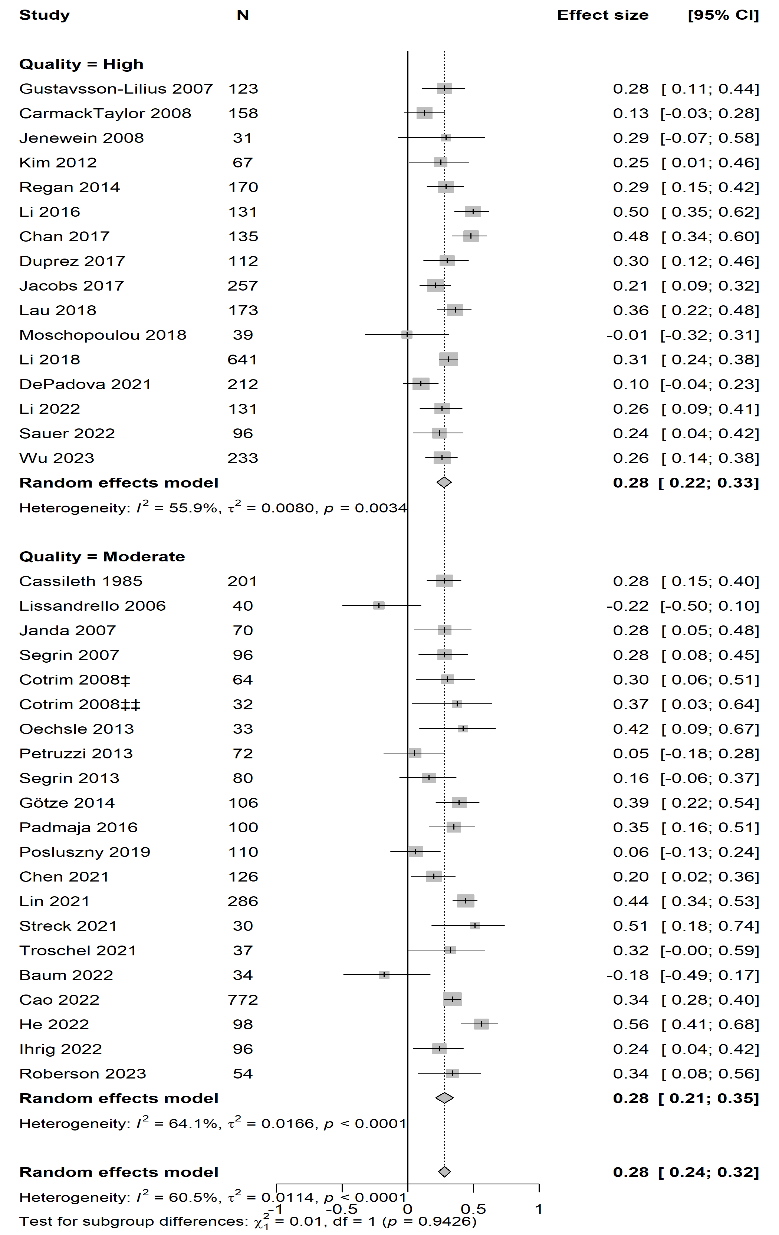 |
| --- | --- |
| C.  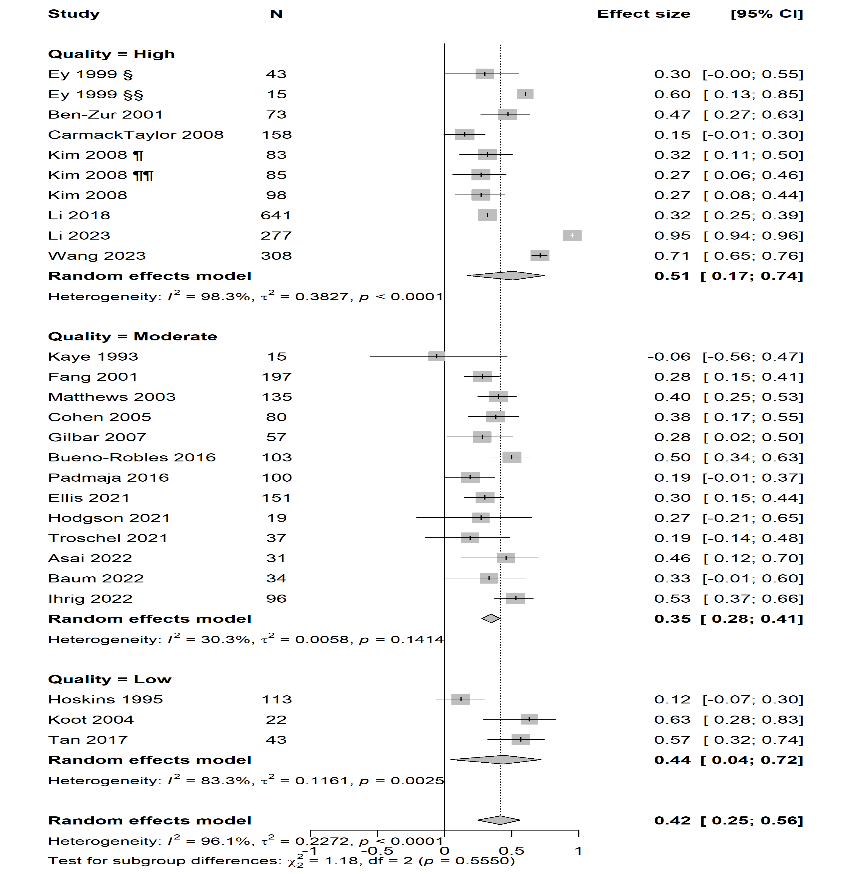 | D.  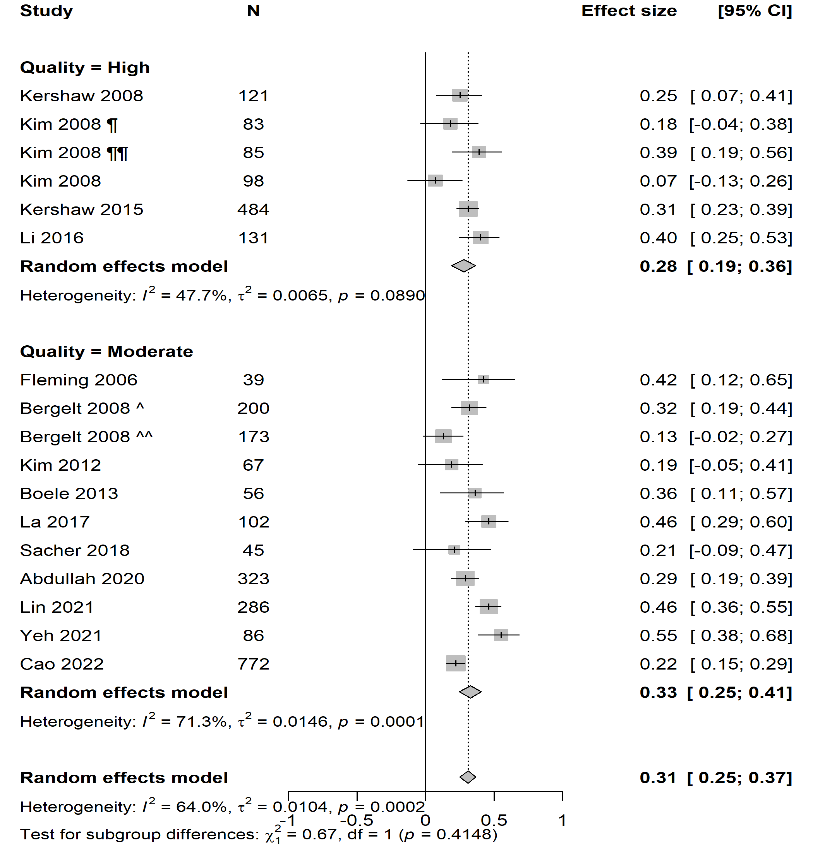 |

†All cancer patient-carer dyads; ‡Dyads with non-stoma patients; ‡‡ Dyads with stoma patients; § Husband carers; §§ Wife carers; ¶Breast cancer; ¶¶Prostate cancer; ^ Female carer; ^^ Male carer.

*Subgroups in which the number of effect sizes <2 were not shown.

**
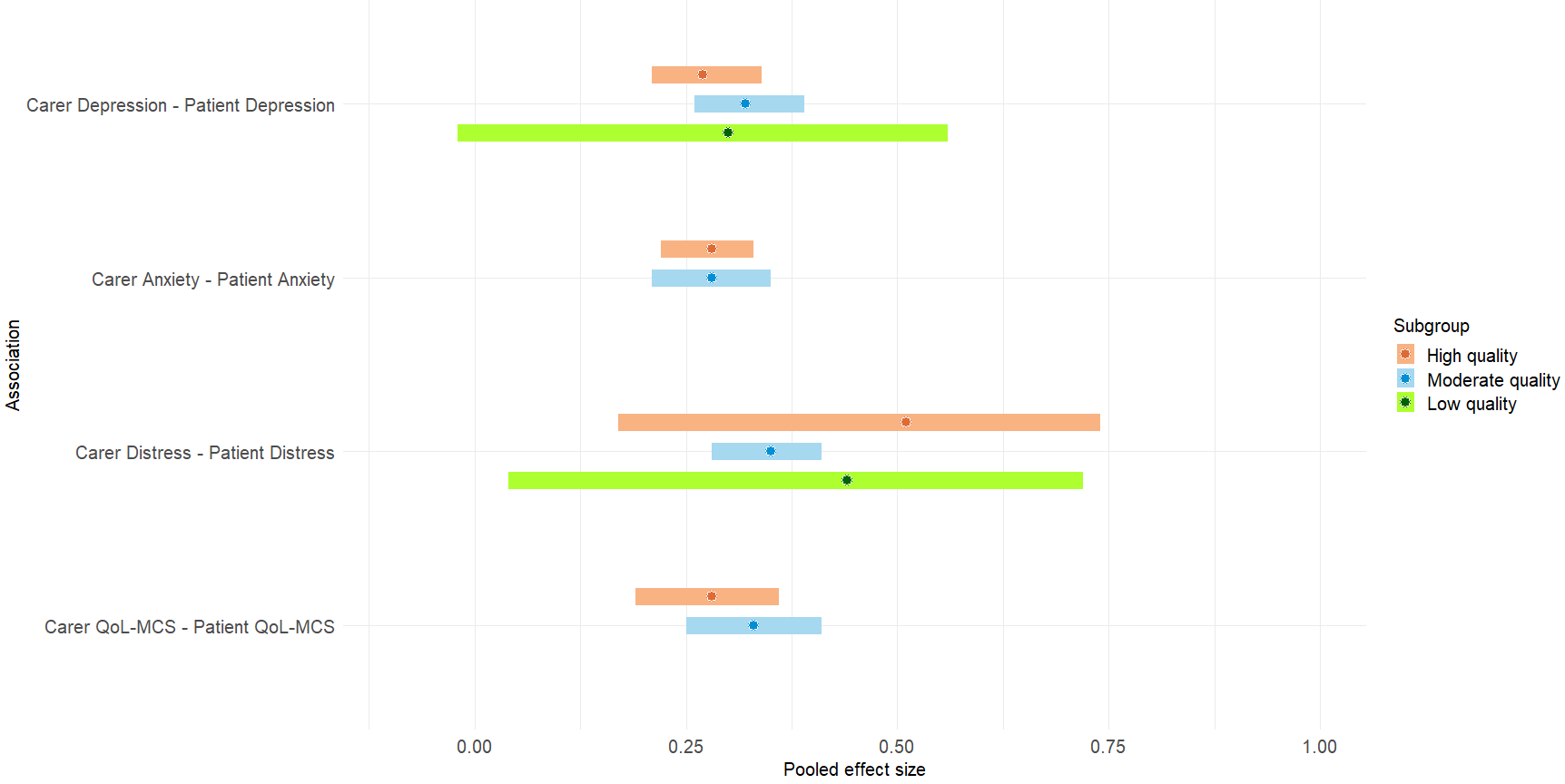
**

*Subgroups in which the number of effect sizes <2 were not shown.
